# Letter to the editor: Reassessing predictive models for hypertension control in resource-constrained settings

**DOI:** 10.1016/j.ijcrp.2025.200531

**Published:** 2025-10-13

**Authors:** Marlon S. Frias, Alwielland Q. Bello

**Affiliations:** a Department of Mathematics, Bukidnon State University, Malaybalay City, 8700, Philippines; b Department of Natural Sciences, Bukidnon State University, Malaybalay City, 8700, Philippines

Dear Editor

We read with great interest the recent article by Azad et al**.** [1] on hypertension control in resource-constrained settings (Int J Cardiol Cardiovasc Risk Prev. 2025; 27:200472). The authors’ integration of conventional statistical approaches with machine learning techniques represents a notable advancement in understanding the multifactorial determinants of blood pressure control in low-resource environments.

The use of multivariate logistic regression to quantify associations between sociodemographic and clinical variables provides a robust baseline for interpreting risk factors [2,3]. However, the study's inclusion of explainable AI models, such as SHAP and LIME, adds interpretability often lacking in traditional predictive frameworks [4]. Importantly, the reported K-Nearest Neighbours (KNN) accuracy of 99 % and mean cross-validation score of 0.984 are impressive, though they invite caution. Such near-perfect performance may indicate potential overfitting, especially in relatively small analytical cohorts (n = 300) [5]. Future studies might benefit from external validation datasets or bootstrapping methods to assess generalizability.

Moreover, the sensitivity analysis identifying socioeconomic variables as dominant predictors underscores the need to integrate social determinants of health into predictive models. Expanding the analysis with hierarchical or mixed-effects modelling could further capture contextual heterogeneity across urban and rural populations.

Overall, this study exemplifies the potential of combining classical biostatistics with interpretable machine learning to inform precision public health. Rigorous validation and replication across diverse settings would strengthen the translational potential of these predictive insights.

## CRediT authorship contribution statement

**Marlon S. Frias:** Conceptualization, Validation, Writing – original draft, Writing – review & editing. **Alwielland Q. Bello:** Writing – original draft, Writing – review & editing.

## Ethical approval

Not required.

## Research registry number

Not applicable.

## Human ethics and consent to participate declarations

Not applicable as no patient data were collected or analyzed in this study.

## Data availability statement

Not applicable as no data were generated or analyzed in this study.

## Clinical trial registration details/number

Not applicable, as this study does not report a clinical trial.

## Generative AI use statement

Generative AI tools, including Paperpal and ChatGPT-4o, were utilized solely for language, grammar, and stylistic refinement. These tools had no role in the conceptualization, data analysis, interpretation of results, or substantive content development of this manuscript. All intellectual contributions, data analysis, and scientific interpretations remain the sole work of the authors. The final content was critically reviewed and edited to ensure accuracy and originality. The authors take full responsibility for the accuracy, originality, and integrity of the work presented.

## Declaration of funding

No funding was received for this study.

## Declaration of interests

The authors declare that they have no competing financial interests or personal relationships that could have appeared to influence the work reported in this paper.
